# Associations between two common single nucleotide polymorphisms (rs2241766 and rs1501299) of ADIPOQ gene and coronary artery disease in type 2 diabetic patients: a systematic review and meta-analysis

**DOI:** 10.18632/oncotarget.18317

**Published:** 2017-05-31

**Authors:** Na Zhao, Ningxia Li, Shengjun Zhang, Qiang Ma, Cong Ma, Xiaolan Yang, Jie Yin, Rui Zhang, Jing Li, Xiaogang Yang, Tao Cui

**Affiliations:** ^1^ Department of Blood Transfusion, The Dongguan District of The Affiliated Hospital of Yan’an University, Yan’an, People’s Republic of China; ^2^ Department of Clinical Laboratory, The Second Affiliated Hospital of Xi’an Medical College, Xi’an, People’s Republic of China; ^3^ Department of General Surgery, The Affiliated Hospital of Yan’an University, Yan’an, People’s Republic of China; ^4^ Department of Vascular Disease and Hypertension, Peripheral Vascular, First Affiliated Hospital of Xi’an Jiaotong University, Xi’an, People’s Republic of China; ^5^ Department of Cardiology, The First Affiliated Hospital of Medical College of Nanchang University, Nanchang, People’s Republic of China; ^6^ Department of Clinical Laboratory, The First Renmin Hospital of Baiyin, Baiyin, People’s Republic of China; ^7^ Department of Clinical Laboratory, The Diagnosis Technology Company of Di’an, Xi’an, People’s Republic of China; ^8^ Department of Clinical Laboratory, The Affiliated Hospital of Yan’an Hospital, Yan’an, People’s Republic of China; ^9^ Department of Infection Control, The Renmin Hospital of Yan’an City, Yan’an, People’s Republic of China; ^10^ Department of Neurosurgery, The Affiliated Hospital of Yan’an University, Yan’an, People’s Republic of China; ^11^ Department of Cardiology, The First Renmin Hospital of Xianyang, Xianyang, People’s Republic of China

**Keywords:** ADIPOQ, polymorphism, coronary artery diseae, type 2 diabetic mellitus

## Abstract

ADIPOQ gene polymorphisms were indicated to be associated with coronary artery disease (CAD) in diabetic patients, however, published studies reported inconsistent results. We performed this meta-analysis to reach a more accurate estimation of the relationship between two common ADIPOQ genetic polymorphisms (rs2241766 and rs1501299) and CAD risk in diabetic patients. Eligible studies were retrieved by searching PubMed, Embase, Wangfang, VIP database and China National Knowledge Infrastructure databases. Included and excluded criteria were formulated. The case group was diabetic patients with CAD, and the control group was diabetic subjects without CAD. Summary odds rations (ORs) and 95% confidence intervals (CIs) were used to evaluate ADIPOQ polymorphisms associations with CAD risk in diabetic group. Heterogeneity was evaluated by Q statistic and I^2^ statistic. A total of twelve published articles, involving 3996 cases and 8876 controls were included in this meta-analysis. The pooled results from rs1501299 polymorphism showed decreased risk in homozygote model (TT VS GG: OR=0.67, 95%CI=0.54-0.83). Heterogeneity was detected in our study. Sensitivity analysis and subgroup analysis were conducted in the meta-analysis. For rs2241766 polymorphism, an increased risk was detected in Caucasian subgroup in heterozygote model (CT VS TT: OR=1.19, 95%CI=1.00-1.42). In genotyping method (PCR-RFLP) subgroup, an increased risk was found in recessive model (GG VS GT+TT: OR=2.05, 95%CI=1.23-3.39). In the sensitivity analysis of rs1501299, decreased risk was detected in allelic model (T VS G: OR=0.86, 95%CI=0.76-0.98) and recessive model (TT VS TG+GG: OR=0.47, 95%CI=0.33-0.67). Publication bias is not observed in our results. Our meta-analysis suggests that the rs1501299 polymorphism may play a protective role in CAD in diabetic patients. The rs2241766 polymorphism is found to be associated with a significant increase in CAD risk in Caucasian and genotyping method (PCR-RFLP) subgroups. Further studies are needed to confirm the prediagnostic effect of the two gene polymorphisms in CAD risk in diabetic patients.

## INTRODUCTION

Type 2 diabetic mellitus (T2DM) is the predominant type of diabetic mellitus which is a complex metabolic disorder with hereditary and environmental factors.[1] Coronary artery disease (CAD) is also a multifactorial and polygenic disorder. The emergency of CAD is two to four times more frequent in diabetic patients than non-diabetic subjects, which implies the hidden mechanism of CAD risk in T2DM groups.[2] The serum levels of adiponectin, an adipocyte-derived hormone, are indicated to be associated with different metabolic syndrome [3], including obesity [4], type 2 diabetes [5], and insulin resistance [6]. And low plasma levels of adiponectin has been shown to be related with CAD risk.[7–9]

The gene coding for adiponectin, ADIPOQ, is located on chromosome 3q27, which is the susceptible locus for CAD and T2DM.[10, 11] A wide range of anti-athergenic effects of adiponectin is reported.[12] Its genetic deficit could increase the risk of CAD in both general population and patients with type 2 diabetes.[13] Since plasma adiponectin level is affected by genetic factors such as single-nucleotide polymorphisms in the adiponectin gene (ADIPOQ), many studies about the associations between the polymorphisms of ADIPOQ and CAD, T2DM, Obesity and insulin resistance have been reported. And the SNPs of the adiponectin gene are reported to be associated with CAD and T2DM.[5, 8, 14]

The polymorphisms of rs2241766 and rs1501299 are two common single nucleotide polymorphisms of ADIPOQ. The rs2241766 polymorphism is a T/G substitution in exon2 and the rs1501299 is a G/T substitution in intron2. The influence of the two SNPs on CAD have been investigated in patients with T2DM, [15–17] the results are inconsistent. In the study of Bacci et al. [18] and Filippi et al.[19], no association was found between the rs2241766 polymorphism and CAD risk in T2DM patients. However, significant association was reported by Ma et al.[20] For the rs1501299 polymorphism, a protective role was reported in the study of QI et al.[21, 22] But an increased risk was reported by Mohammadzadeh et al.[23] The small numbers and various populations of the published studies may partially account for the controversial results. Our meta-analysis therefore aims to pool current evidence together for better understanding of the potential associations between the rs2241766 and rs1501299 polymorphisms and CAD risk in type 2 diabetic patients.

## RESULTS

### The characteristics of the include studies

214 articles were obtained by online and manual search. After removing duplicates and screening title and abstract, eighty articles were included. Seven articles were excluded for lack of detailed genotype distribution. Finally, a total of twelve published articles, [11, 18, 20–23, 29–34] involving 3996 cases and 8876 controls were included in this meta-analysis (Seen in the Supplementary Table 2).

The characteristics of all the included articles are summarized in Table 1. For rs2241766, twelve studies are included with 2136 cases and 3391 controls; ten studies with 1860 cases and 5485 controls are included for rs1501299.

**Table 1:** Characteristics of included studies selected for meta-analysis

Study	Year	Country	Enthicity	Region	Sample size		Genotype distributions in cases/controls			Genotyping	Quality	HWE*
					Case	Control	11	12	22	method	score	
rs2241766												
Mohammadzadeh	2016	Iran	Caucasian	Western Asian	100	100	75/65	24/31	1/4	PCR-RFLP	8	0.899
Mofarrah	2016	Iran	Caucasian	Western Asian	152	72	82/56	35/13	35/3	PCR-HRM	8	0.072
Esteghamati	2012	Iran	Caucasian	Western Asian	114	127	48/68	41/46	25/13	PCR-RFLP	7	0.222
Nan	2012	China	Mongoloid	Eastern Asian	213	467	115/237	84/191	14/39	PCR-HRM	8	0.952
Al-Daghri	2011	Saudi Arabia	Caucasian	Western Asian	122	298	77/220	35/72	10/6	PCR-RFLP	7	0.969
Chiodini	2010	Italy	Caucasian	Southern Europe	499	503	321/359	168/126	10/18	Taqman	8	0.102
Ma	2008	China	Mongoloid	Eastern Asian	159	31	94/12	55/16	10/3	PCR-RFLP	8	0.479
Qi	2006	China	Mongoloid	Eastern Asian	266	672	204/529	62/143		Taqman	8	-
Qi	2005	China	Mongoloid	Eastern Asian	219	599	170/440	49/159		Taqman	8	-
Lacquemant Swiss	2004	Switzerland	Caucasian	Northern Europe	107	181	76/145	27/34	4/2	PCR	8	0.997
Lacquemant French	2004	Switzerland	Caucasian	Northern Europe	55	130	27/57	25/65	3/8	PCR	7	0.059
Bacci	2004	Italy	Caucasian	Southern Europe	130	211	90/140	35/60	5/11	PCR	8	0.182
												
rs1501299												
Mohammadzadeh	2016	Iran	Caucasian	Western Asian	100	100	38/56	55/42	7/2	PCR-RFLP	9	0.063
Katakami	2012	Japan	Mongoloid	Eastern Asian	213	2637	129/1358	71/1047	13/232	PCR	8	0.139
Esteghamati	2012	Iran	Caucasian	Western Asian	114	127	76/63	30/47	8/17	PCR	8	0.095
Al-Daghri	2011	Saudi Arabia	Caucasian	Western Asian	123	297	47/111	57/142	19/44	PCR-RFLP	7	0.897
Chiodini	2010	Italy	Caucasian	Southern Europe	499	503	271/239	189/198	39/66	Taqman	8	0.016
Qi	2006	China	Mongoloid	Eastern Asian	280	684	159/374	104/258	17/52	Taqman	8	0.420
Qi	2005	China	Mongoloid	Eastern Asian	228	594	105/293	111/249	12/52	Taqman	8	0.930
Lacquemant Swiss	2004	Switzerland	Caucasian	Southern Europe	106	179	57/96	40/65	9/18	PCR	7	0.166
Lacquemant French	2004	Switzerland	Caucasian	Northern Europe	55	130	25/73	26/50	4/7	PCR	8	0.679
Bacci	2004	Italy	Caucasian	Southern Europe	142	234	70/118	65/83	7/28	PCR	7	0.031

For rs2241766 variant, 11, 12 and 22 represent TT, GT, GG, respectively; for rs1501299 variant, 11, 12 and 22 represent GG, TG and TT, respectively.

* *P* value for Hardy–Weinberg equilibrium test in controls

### Meta-analysis results

#### ADIPOQ rs2241766 polymorphism and CAD risk in T2DM patients

**Figure 1:**
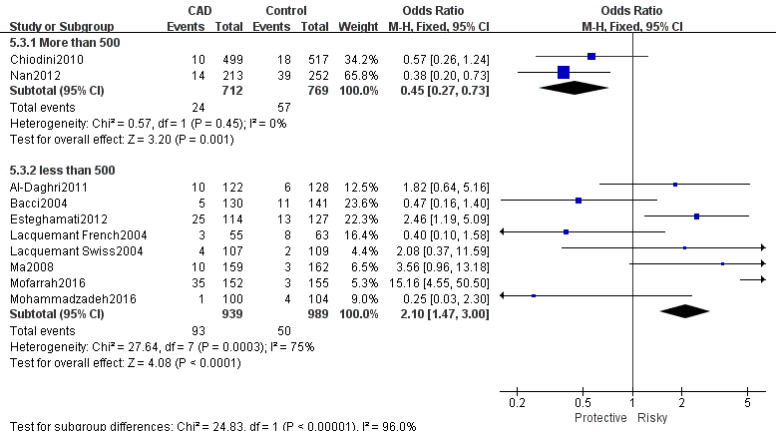
Forest plot of CAD risk associated with the GG genotype in ADIPOQ rs2241766 polymorphism OR = odd ration, CI = confidence interval.

**Figure 2:**
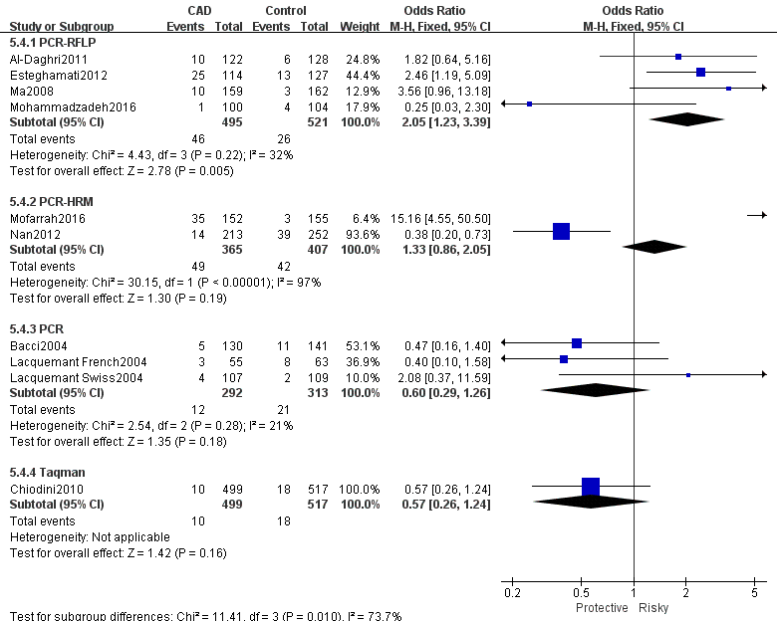
Forest plot of CAD risk associated with the T allele compared with the G allele in ADIPOQ rs1501299 polymorphism OR = odd ration, CI = confidence interval.

**Figure 3:**
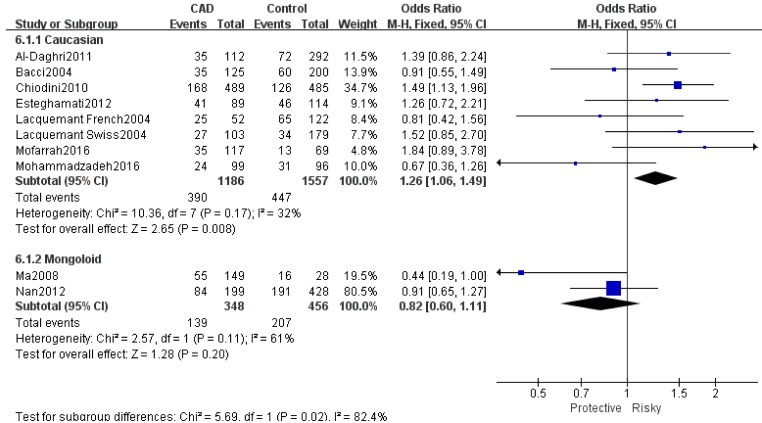
Forest plot for rs2241766 GT *VS* GG Ethnicity OR = odd ration, CI = confidence interval.

Table 2 showed the results of this meta-analysis for ADIPOQ rs2241766 polymorphism and CAD in diabetic patients. The rs2241766 polymorphism showed no significant associations with CAD in type 2 diabetic group based on combined results from all studies. Besides, significant heterogeneity was found. Sensitivity analysis was performed by sequentially omitting 1 individual study at a time, but heterogeneity was still significant in five genetic models. Subgroup analysis was introduced for further study, and significant associations were found in different subgroups. Our results showed that rs2241766 polymorphism was associated with decreased risk of CAD in recessive model (GG VS GT+TT: OR=0.45,95%CI=0.27-0.73,P_h_=0.001) in the subgroup of sample size(more than 500)(Figure 1), but increased risk of CAD was found in the subgroup of genotyping method(PCR-RFLP) in recessive model (GG VS GT+TT: OR=2.05, 95%CI=1.23-3.39, P_h_=0.005)(Figure 2). In the Caucasian subgroup, increased risk was also detected in heterozygote model (CT VS TT: OR=1.19, 95%CI=1.00-1.42, Ph=0.89) (Figure 3).

**Table 2:** Meta-analysis results of the associations between rs2241766 and rs1501299 polymorphisms in ADIPOQ gene and CAD risk in T2DM patients

Genetic model	NO. of studies	OR[95%CI]	P_meta-analysis_	Bon	FDR	*I*^2^ (%)	P^a^_heterogeneity_	Statistical method
rs2241766								
G VS T	12	1.18 [0.89, 1.56]	0.260	1.000	0.613	80.00	0.000	Random
GG VS GT+TT	12	1.21 [0.56, 2.61]	0.630	1.000	0.630	81.00	0.000	Random
GG+GT VS TT	12	1.11 [0.88, 1.39]	0.390	1.000	0.613	68.00	0.000	Random
GT VS TT	12	1.08 [0.86, 1.37]	0.490	1.000	0.613	52.00	0.030	Random
GG VS TT	12	1.33 [0.69, 2.55]	0.400	1.000	0.613	72.00	0.000	Random
rs1501299								
T VS G	10	**0.86 [0.76, 0.98]**	**0.020**	0.100	**0.033**	46.00	0.060	Fixed
TT VS TG+GG	10	0.40 [0.22, 0.71]	0.002	0.010	0.005	84.00	0.000	Random
TT+TG VS GG	10	0.95 [0.78, 1.16]	0.600	1.000	0.600	63.00	0.003	Random
TG VS GG	10	0.95 [0.79, 1.13]	0.530	1.000	0.600	45.00	0.070	Fixed
TT VS GG	10	**0.67 [0.54, 0.83]**	**0.000**	**0.002**	**0.002**	36.00	0.120	Fixed

OR= odd ratio; CI= confidence interval.

^a^ *P* value for between-study heterogeneity based on Q test;Bon= p value in Bonferroni test; FDR= false discovery rate.

Significant results are marked in bold.

#### ADIPOQ rs1501299 polymorphism and CAD risk in T2DM patients

**Figure 4:**
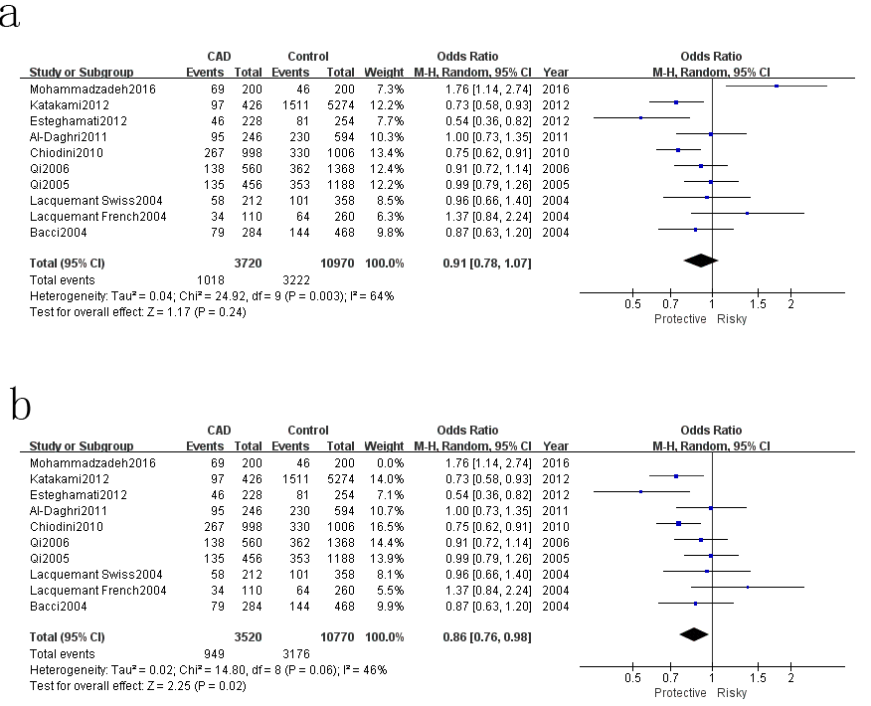
Forest plot for rs1501299 T *VS* G **a**. Pooled results, **b**. results move out Mohammadzadeh 2016.

**Figure 5:**
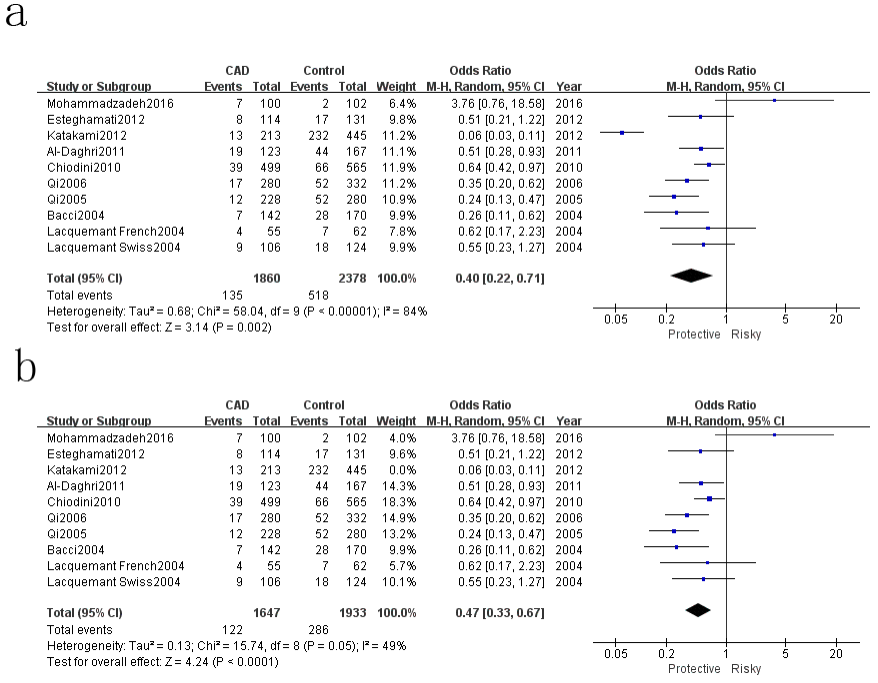
Forest plot for rs1501299 TT VS TG+GG **a**. Pooled results, **b**. results move out Katakami 2012.

The results of associations between ADIPOQ rs150299 polymorphism and CAD risk in type 2 diabetic subjects were showed in Table 3. A decreased risk of rs1501299 was only detected in homozygote model (TT VS GG: OR=0.67, 95%CI=0.54-0.83, Ph=0.0003), and significant heterogeneity were found in the other four genetic models. Therefore, sensitivity analysis was conducted to explore the source of heterogeneity. Heterogeneity were significant decreased in the allelic model, dominant model and heterozygote model when Mohammadzadeh et al.[23] was moved out. A decreased risk was detected in allelic model (T VS G: OR=0.86, 95%CI=0.76-0.98, Ph=0.02) (Figure 4). Besides, a decreased risk was also found in recessive model (TT VS TG+GG: OR=0.47, 95%CI=0.33-0.67, Ph=0.000) (Figure 5) when Katakami et al.[32] was moved out.

**Table 3:** Results of subgroup analysis for ADPIOQ rs2241766 polymorphisms and CAD in type 2 diabetic patients

		G VS T		GG+GT VS TT		GG VS GT+TT		GT VS TT		GG VS TT	
Study group	Study numbers	OR[95%CI]	I2(%)/Ph	OR[95%CI]	I2/Ph	OR[95%CI]	I2/Ph	OR[95%CI]	I2/Ph	OR[95%CI]	I2/Ph
**Total**	10	1.18[0.89,1.56]	80/0.000	1.11[0.88,1.39]	68/0.000	1.21[0.56,2.61]	81/0.000	1.14[0.98,1.32]	52/0.030	1.33[0.69,2.55]	72/0.000
**Enthicity**											
Caucasian	8	1.32[1.15,1.51]	78/0.000	1.30[0.97,1.73]	64/0.007	1.57[1.13,2.18]	79/0.000	1.26[1.06,1.49]	32/0.17	1.74[1.23,2.46]	72/0.000
Mongoloid	2	0.82[0.65,1.04]	49/0.160	0.86[0.66,1.13]	43/0.15	0.63[0.37,1.06]	89/0.003	0.82[0.60,1.11]	61/0.11	0.68[0.38,1.25]	0/0.490
**Region**											
Eastern Asian	2	0.82[0.65,1.04]	49/0.160	0.89[0.73,1.07]	43/0.15	0.63[0.37,1.06]	89/0.003	0.82[0.60,1.11]	61/0.110	0.67[0.37,1.21]	0/0.49
Western Asian	4	1.66[1.34,2.06]	85/0.000	1.51[1.16,1.96]	76/0.005	3.27[2.04,5.22]	77/0.004	1.21[0.91,1.62]	40/0.17	2.97[1.10,8.03]	65/0.040
Southern Europe	2	1.11[0.91,1.36]	49/0.160	1.24[0.98,1.56]	64/0.10	0.53[0.28,1.00]	0/0.790	1.32[1.04,1.68]	66/0.09	0.65[0.34,1.23]	0/0.85
Northern Europe	2	1.19[0.84,1.68]	70/0.07	1.20[0.79,1.82]	63/0.10	0.75[0.28,2.05]	54/0.140	1.15[0.75,1.76]	50/0.16	1.60[0.35,7.42]	48/0.16
**Sample Size**											
More than 500	2	1.05[0.88,1.25]	69/0.07	1.07[0.91,1.25]	60/0.060	**0.45[0.27,0.73]**	**0/0.450**	1.22[0.99,1.51]	80/0.030	0.69[0.42,1.14]	0/0.74
less than 500	8	1.29[1.10,1.51]	82/0.000	1.21[0.99,1.46]	73/0.000	2.10[1.47,3.00]	75/0.000	1.06[0.87,1.31]	45/0.080	2.07[1.41,3.04]	69/0.002
**Genotyping Method**											
PCR-RFLP	4	1.04[0.58,1.87]	85/0.000	1.12[0.85,1.47]	78/0.003	**2.05[1.23,3.39]**	**32/0.22**	0.99[0.74,1.33]	62/0.050	1.96[1.15,3.32]	74/0.008
PCR-HRM	2	1.71[0.45,6.59]	95/0.000	1.15[0.87,1.53]	91/0.000	1.33[0.86,2.05]	97/0.000	1.04[0.77,1.41]	67/0.080	1.55[0.95,2.54]	92/0.000
PCR	3	1.06[0.70,1.61]	58/0.09	1.05[0.77,1.43]	46/0.160	0.60[0.29,1.26]	21/0.28	1.04[0.75,1.43]	20/0.290	1.03[0.50,2.16]	29/0.24
Taqman	1	1.21[0.96,1.52]	#	1.13[0.95,1.36]	65/0.060	0.57[0.26,1.24]	#	1.49[1.13,1.96]	#	0.62[0.28,1.37]	#

Abbreviations: CI, confidence interval; OR, odds ratio; Ph, P value for heterogeneity from Q-test; I2, the proportion of the total variation across studies due to heterogeneity.

#No heterogeneity was observed for only one study.

Significant results are marked in bold.

### Publication bias

No publication bias was detected among studies regarding the association between the rs1501299 and rs2241766 polymorphisms (P_rs1501299_=0.088; P_rs2241766_= 0.799) and CAD risk in diabetic patients (Figure 7, 8).

**Figure 6:**
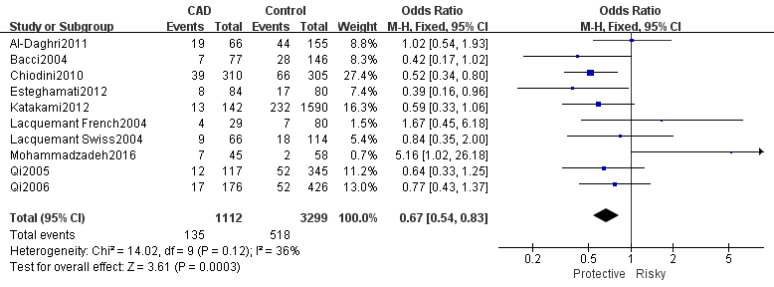
Forest plot for rs1501299 TT VS GG total

**Figure 7:**
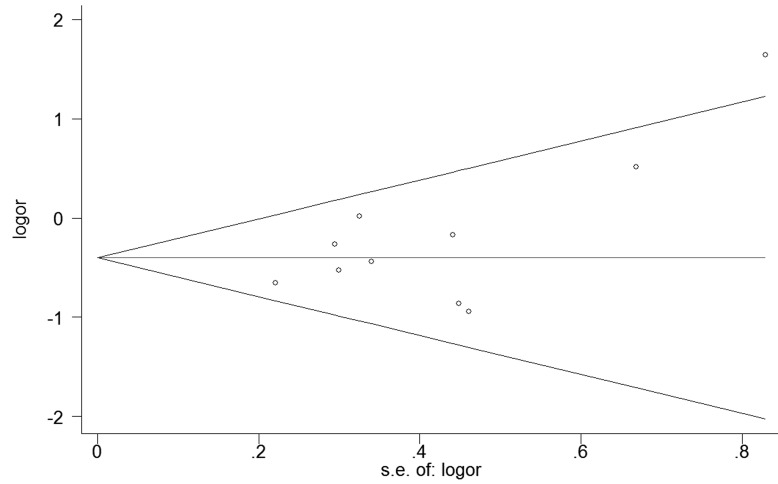
Begg's funnel plot of rs1501299 for contrast in overall analysis in recessive model Each point represents a separate study for the indicated association. Size graph symbol by weighs. Log [OR] natural logarithm of OR. Horizontal line means effect size.

**Figure 8:**
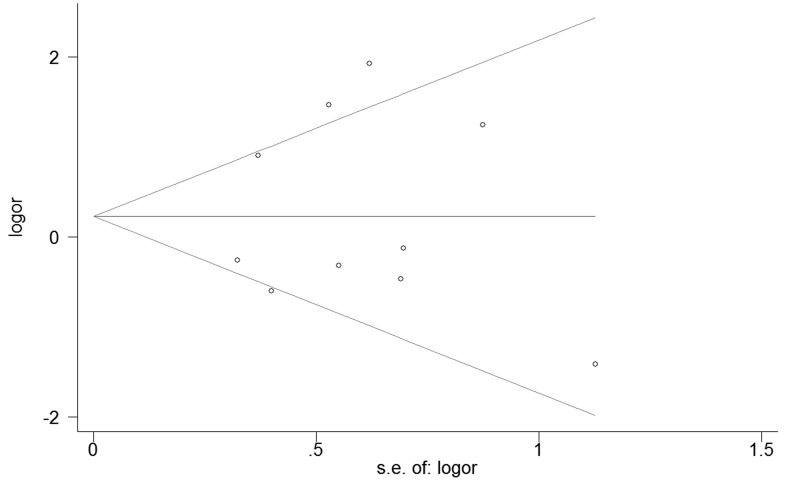
Begg's funnel plot of rs2241766 for contrast in overall analysis in recessive model Each point represents a separate study for the indicated association. Size graph symbol by weighs. Log [OR] natural logarithm of OR. Horizontal line means effect size.

## DISCUSSION

Type 2 diabetes is associated with a marked increase in the risk of coronary artery disease.[16] In this present meta-analysis, which included 3996 cases and 8876 controls, the associations between the polymorphisms of ADIPOQ rs2241766 and rs1501299 and CAD risk in type 2 diabetic patients were analyzed. The results showed that the ADIPOQ rs2241766 polymorphism is a risk factor for developing CAD in the subgroup analysis. For rs1501299 polymorphism, a protective role of CAD in T2DM was found in different genetic models. Several meta-analysis on the association of adiponectin polymorphisms (rs2241766, rs1501299) with CAD in general population suggested that both rs2241766 and rs1501299 were significant high risk factor for CAD.[35–38] However, a low risk of rs1501299 for the development of CAD with type 2 diabetic patients was reported by Sun et al.[39], which was in accordance with our results.

Significant associations between rs2241766 polymorphism and CAD with T2DM were found in different subgroup. In the Caucasian subgroup, a 26% increased risk was detected in GT genotype, which suggests a possible contribution of ethnicity in genetic background for the development of CAD. A high risk of GG genotype in recessive genetic model was observed in the subgroup of genotyping method (PCR-RFLP), which implies different genotyping method may have an influence on the accuracy of genetic analysis. In the subgroup of sample size (more than 500), a relative low risk of GG genotype was detected, which needed further research to confer whether the sample size may have an effect on associations between rs2241766 polymorphism and CAD with T2DM. In the study by Mofarrah and colleagues, rs2241766 of ADIPOQ had a significant association with CAD and G allele carriers had an increased adjusted risk ratio of developing CAD.[30] Besides, rs2241766 polymorphism was significantly associated with the incidence of CAD in T2DM and it could independently alter the CAD risk in diabetic patients based on its strong association even after adjusting for several classical CAD risk factors.[11] Although evidences on increased risk between rs2241766 and CAD with T2DM had emerged, the underlying molecular mechanism was still unknown. The rs2241766 polymorphism located in exon 2 results in a synonymous change (G15G), and is relatively close to exon-intron boundary which may affect the splicing machinery.[31] There is increasing evidence that even silent mutations in coding regions might modify RNA levels by affecting splicing and thus decreasing expression of the gene.[40] In this regard, a bioinformatics analysis revealed a consensus sequence recognized by a functional exonic-splicing-enhancer (ESE) and 87% matched to a sequence at 4 bp in 3’ from rs2241766 polymorphism.[40] Therefore, the rs2241766 might have an influence on the expression of adiponectin gene and associate with CAD risk in T2DM subjects.

**Table 4:** Results of sensitivity analysis of meta-analysis for ADPIOQ rs1501299 polymorphisms and CAD in type 2 diabetic patients

	T VS G		TT+TG VS GG		TT VS TG+GG		GT VS GG		TT VS GG	
Study group 2	OR[95%CI]	I2(%)/P_h_	OR[95%CI]	I^2^/P_h_	OR[95%CI]	I^2^/P_h_	OR[95%CI]	I^2^/P_h_	OR[95%CI]	I^2^/P_h_
Total	0.91[0.78,1.07]	64/0.003	0.95[0.78,1.16]	63/0.004	0.40[0.22,0.71]	84/0.000	1.00[0.82,1.21]	56/0.020	0.67[0.54,0.83]	36/0.120
Mohammadzadeh2016	**0.86[0.76,0.98]**	**46/0.06**	0.89[0.75,1.05]	49/0.05	0.34[0.19,0.60]	84/0.000	0.95[0.79,1.13]	45/0.070	&	&
Katakami2012	0.94[0.79,1.12]	64/0.04	0.99[0.80,1.23]	61/0.009	0.39[0.21,0.73]	86/0.000	1.05[0.86,1.27]	50/0.040	&	&
Esteghamati2012	0.94[0.81,1.10]	59/0.01	0.99[0.82,1.20]	58/0.02	**0.47[0.33,0.67]**	**49/0.050**	1.04[0.86,1.25]	50/0.040	&	&
Al-Daghri2011	0.90[0.76,1.07]	67/0.002	0.95[0.76,1.18]	67/0.002	0.39[0.20,0.74]	86/0.000	1.00[0.81,1.24]	61/0.009	&	&
Chiodini2010	0.94[0.79,1.12]	63/0.006	0.98[0.78,1.23]	64/0.005	0.37[0.20,0.71]	83/0.000	1.03[0.82,1.28]	58/0.010	&	&
Qi2006	0.92[0.76,1.10]	68/0.002	0.96[0.76,1.21]	67/0.002	0.41[0.21,0.79]	86/0.000	1.01[0.81,1.26]	61/0.009	&	&
Qi2005	0.90[0.76,1.08]	66/0.003	0.93[0.74,1.15]	63/0.005	0.42[0.22,0.81]	86/0.000	0.97[0.79,1.19]	54/0.020	&	&
Lacquemant Swiss2004	0.91[0.77,1.08]	68/0.002	0.95[0.76,1.17]	67/0.002	0.38[0.21,0.72]	86/0.000	1.00[0.81,1.23]	61/0.009	&	&
Lacquemant French2004	0.89[0.76,1.04]	63/0.006	0.94[0.76,1.17]	66/0.002	0.38[0.21,0.71]	86/0.000	0.97[0.80,1.18]	57/0.020	&	&
Bacci2004	0.92[0.77,1.09]	68/0.002	0.92[0.75,1.12]	63/0.006	0.42[0.22,0.78]	86/0.000	0.97[0.79,1.20]	58/0.010	&	&

Abbreviations: Ph, P value for heterogeneity from Q-test; I2, the proportion of the total variation across studies due to heterogeneity; CI, confidence interval; OR, odds ratio.

&No heterogeneity was observed in the homozygote model.

Significant results are marked in bold.

For the rs1501299 polymorphism, pooled result was only significant in homozygote model, which implied a 33% decreased risk of TT genotype in the developing of CAD in T2DM subjects. When the study of Mohammadzadeh et al.[23] was removed, the T allele carriers had a 14% decreased risk compared to G allele carriers. Besides, the TT genotype had a 53% decreased risk of developing CAD in T2DM patients in recessive model, if the research of Katakami et al. was moved out. The protective role of rs1501299 polymorphism is consistent to most published results. The G allele of the ADIPOQ rs1501299 is a susceptibility allele for CAD in Japanese type 2 diabetic patients reported by Katakami et al.[32]. A protective role of T allele of rs1501299 polymorphism in developing CAD in Iranian T2DM subjects was also reported by Esteghamati et al.[33]. Moreover, gender was also an important factor in the associations between rs1501299 polymorphism and CAD risk in T2DM. In the study if Qi et al.[21], the homozygous allele T at rs1501299 polymorphism was associated with a significantly lower risk of CAD in diabetic men. However, the frequency of T allele at position rs1501299 in female CAD patients was higher than that of T2DM patients without CAD. In different region, not only in Asian but also in Europe, T allele as an antiatherosclerotic phenotype was also widely observed, more research from other region were needed to support the result. The same as rs2241766 polymorphism, molecular mechanism of rs1501299 was hardly known. Although rs1501299 is located in an intronic region with no apparent biological function, this SNP may affect the expression level of the gene through some unknown mechanisms, or it may be in LD with undiscovered SNPs in the ADIPOQ gene or other genes with biological effects on insulin resistant.[17]

In haplotype analysis, the haplotypes 45T-276T and 45G-276T in compared to the referent haplotype 45T-276G was indicated a protective effect reported by Esteghamati et al.[33], however, in the study of Mohammadzadeh et al.[23], the haplotype 45G-276G indicated a protective effect against the presence of CAD in T2DM patients as compared to the reference 45T-276G haplotype. The differences in haplotypes might shed a new light on interactions between genes and epigenetic modifications.

There were several limitations in this meta-analysis. First, only English and Chinese articles were included in our study, which thus may bias the results. Second, patient heterogeneity and confounding factors might have distorted the analysis. Third, there was significant heterogeneity in some of the pooled analyses, which may have affected the meta-analysis results, even though we adopted the random effects model. Fourth, the number of included studies was relatively small in some subgroups, thus results should be interpreted with caution. In addition, the potential influence on genotype-CAD associations by environment factors is worthy of consideration.

In conclusion, Our meta-analysis suggests that the rs1501299 polymorphism may play a protective role in CAD, and the possible protective role in T allele and TT genotype in CAD patients with T2DM needs more researches. The rs2241766 polymorphism is found to be associated with a significant increase in CAD risk in Caucasian subgroup based on our analysis. Further studies are needed to confirm the prediagnostic effect of ADIPOQ gene polymorphisms in CAD risk in diabetic patients.

## MATERIALS AND METHODS

The systematic review was written in adherence to the PRISMA (Preferred Reporting Items for Systematic Reviews and Meta-analyses) checklist.[24] Ethical approval was not necessary according to local legislation because of the type of study (meta-analysis).[25]

### Identification of the related studies

Embase, PubMed, Wangfang, VIP databases and China National Knowledge Infrastructure databases were thoroughly searched in June 2016 by the first two investigators to identify potential studies addressing the associations between the ADIPOQ polymorphisms and coronary artery disease. The terms “coronary artery disease,” “coronary heart disease,” “ADIPOQ,” “APM,” “diabetic mellitus,” “T2DM,” “variant,” “polymorphism,” and “polymorphisms” were used. The missing data (the data that we failed to identify during the electronic search) were obtained by reviewing the citations of review articles and all eligible studies.

### Inclusion and exclusion criteria

Studies in the meta-analysis must meet the following inclusion criteria: (1) evaluation of the association between ADIPOQ polymorphisms and coronary artery disease in diabetic patients. Subjects with T2DM were based on American Diabetes Association (ADA). The case group was T2DM patients with CAD, the criteria for CAD, conformed by coronary angiography, was ≥50% stenosis of at least one segment of a major coronary artery or its main branches. The control group consisted of T2DM subjects with normal exercise tolerance test and negative history of CAD; (2) case-control study or cohort design; (3) detailed genotype data could be acquired to calculate odds ratios (ORs) and 95% confidence intervals (CIs); Exclusion criteria: (1) duplication of previous publications; (2) comment, review and editorial; (3) study with no detailed genotype data. The selection of the studies was achieved by two investigators independently, according to the inclusion and exclusion criteria by screening the title, abstract and full-text. Any dispute was solved by discussion.

### Data extraction

From each study, the following data were independently extracted by the first two investigators using a standardized form: first author's last name, year of publication, study country, ethnicity, region, genotyping methods, Hardy–Weinberg equilibrium, number of cases and controls, genotype distribution in cases and controls for ADIPOQ, quality score. Different ethnicity descents were classified as Caucasian and Mongoloid. Disagreements were resolved through discussion with a 3rd investigator.

### Quality assessment

The first two authors independently assessed the quality of the included studies, according to a set of criteria (Supplementary Table 1) modified on the basis of the Newcastle-Ottawa quality assessment scale. Scores ranged from 0 to 10, with 0 as the lowest and 10 as the highest quality.

### Statistics analysis

Hardy–Weinberg equilibrium (HWE) was evaluated for each study by Chi-square test in control groups, and P < 0.05 was considered as a significant departure from HWE. Odds ratio (OR) and 95% confidence intervals (CIs) were calculated to evaluate the strength of the association between ADIPOQ polymorphisms and CAD in T2DM subjects. Pooled ORs were performed for allelic model (rs2241766: G versus T; rs1501299: T versus G), heterozygote model (rs2241766: GT versus TT; rs1501299: TG versus GG), homozygote model (rs2241766: GG versus TT; rs1501299: TT versus GG), dominant model (rs2241766: GG+GT versus TT; rs1501299: TT+TG versus GG), recessive model (rs2241766: GG versus GT+TT; rs1501299: TT versus TG+GG), respectively. The statistically significant level was determined by Z-test with P value less than 0.05. Heterogeneity was evaluated by Q statistic (significance level of P < 0.1) and I^2^ statistic (greater than 50% as evidence of significant inconsistency). To adjust for multiple comparisons, we applied the Bonferroni method to control the false discovery rate (FDR) [26, 27].

Heterogeneity between studies was evaluated with the I^2^ test, and a higher I^2^ values means higher levels of heterogeneity (I^2^ >90%: extreme heterogeneity; I^2^ = 70% to 90%: large heterogeneity; I^2^ =50% to 70%: moderate heterogeneity; I^2^ < 50%: no heterogeneity). In heterogeneity evaluation, when the I^2^ < 50%, the fixed-effects model would be used; if the I^2^ =50% to 90%, a random-effects model was used; if the I^2^ > 90%, the studies would not be pooled.[28] If heterogeneity was significant, sensitivity analysis was performed to detect the heterogeneity by omitting each study in each turn. Besides, subgroup analyses were stratified by ethnicity (Caucasian, Mongoloid), region (Eastern Asian, West Asian, Southern Europe, North Europe), sample size(more than 500, less than 500), genotyping method(PCR-RFLP, PCR-HRM, PCR, Taqman). The publication bias was assessed with Begg's funnel plot and Egg's test. Review Manager, Version 5.3 (The Nordic Cochrane Centre, The Cochrane Collaboration; Copenhagen, Denmark) was used for all analysis.

## SUPPLEMENTARY MATERIALS FIGURES AND TABLE






