# Supplementary material for: Associations between two common single nucleotide polymorphisms (rs2241766 and rs1501299) of ADIPOQ gene and coronary artery disease in type 2 diabetic patients: a systematic review and meta-analysis
Source: Oncotarget. 2017 May 31;8(31):51994–2005. doi: 10.18632/oncotarget.18317 (PMC5584307; doi:10.18632/oncotarget.18317)
Supplement: Supplementary file 1 [file oncotarget-08-51994-s001.doc]

| **Table S1.** **Scale for methodological quality assessment.** | |
| --- | --- |
| Criteria | Score |
| 1.Representativeness of cases |  |
| CAD and T2DM diagnosed according to acknowledged criteria. | 2 |
| Mentioned the diagnosed criteria but not specifically described. | 1 |
| Not Mentioned. | 0 |
| 2.Source of controls |  |
| Population or community based | 3 |
| Hospital-based CAD-free controls | 2 |
| CAD-free controls with related diseases | 1 |
| Not described | 0 |
| 3.Sample size |  |
| >500 | 2 |
| 300-500 | 1 |
| <300 | 0 |
| 4.Quality control of genotyping methods |  |
| Repetition of partial/total tested samples with a different method | 2 |
| Repetition of partial/total tested samples with the same method | 1 |
| Not described | 0 |
| 5.Hardy-Weinberg equilibrium (HWE) |  |
| Hardy-Weinberg equilibrium in control subjects | 1 |
| Hardy-Weinberg disequilibrium in control subjects | 0 |
