# Supplementary material for: Associations between two common single nucleotide polymorphisms (rs2241766 and rs1501299) of ADIPOQ gene and coronary artery disease in type 2 diabetic patients: a systematic review and meta-analysis
Source: Oncotarget. 2017 May 31;8(31):51994–2005. doi: 10.18632/oncotarget.18317 (PMC5584307; doi:10.18632/oncotarget.18317)
Supplement: Supplementary file 2 [file oncotarget-08-51994-s002.doc]

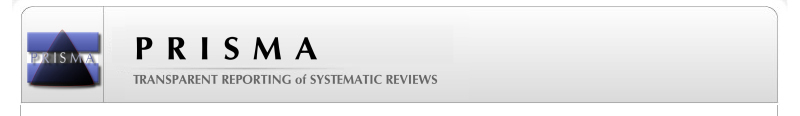
**PRISMA 2009 Flow Diagram**

**Screening**

**Included**

**Eligibility**

**Identification**

Records identified through PubMed and Embase database searching
(n = 183)

Additional records identified through, VIP, Wangfang, and CNKI database searching
(n = 31)

Records after duplicates removed
(n = 80)

Title and abstract screened
(n = 23)

Title and abstract excluded
(n = 59)

Full-text articles assessed for eligibility
(n = 18)

Full-text articles excluded,

Reviews, editorial

(n = 5)

Studies included in qualitative synthesis
(n = 11)

Studies included in quantitative synthesis (meta-analysis)
(n = 12)

Full-text articles excluded,

Lack of detailed genotype distribution data
(n = 7)
